# The state of wildfire and health research: emerging trends, challenges and gaps

**DOI:** 10.1093/inthealth/ihaf032

**Published:** 2025-04-08

**Authors:** Ana Raquel Nunes

**Affiliations:** Warwick Medical School, University of Warwick, Coventry CV4 7AL, UK

**Keywords:** bibliometric analysis, exposure, future directions, human health, research trends, wildfire

## Abstract

**Background:**

The increasing frequency and severity of wildfires, exacerbated by climate change, population growth and land use changes, have escalated public health risks. These events are associated with respiratory and cardiovascular diseases and adverse mental health outcomes. Vulnerable populations, including children, older people and those with pre-existing health conditions, face particularly high risks.

**Methods:**

This study evaluates the existing literature on wildfire-related health impacts. Key variables include publication frequency, geographic distribution, collaborative networks and funding patterns.

**Results:**

Findings reveal a concentration of research in high-income regions, particularly North America and Europe, with limited studies from wildfire-prone but under-represented areas such as Latin America, Oceania, Africa and the Caribbean. This geographical disparity restricts comprehensive understanding and effective public health responses to wildfire impacts. The analysis also underscores the need for interdisciplinary approaches.

**Conclusions:**

Wildfires continue to pose significant global public health challenges. There is a critical need for more inclusive research efforts, enhanced international collaboration and a stronger focus on health-specific outcomes, especially in under-represented regions. Expanding research in these areas is essential to inform effective public health policies and interventions that address the health risks posed by wildfires worldwide.

## Introduction

Wildfires have emerged as a critical global concern, driven by the dual pressures of climate change and human activity.^1–7^ These natural events not only devastate ecosystems but also pose significant risks to human health,^8–10^ primarily through the inhalation of wildfire smoke,^11,12^ which contains hazardous pollution such as particle matter (PM 2.5), nitrogen dioxide (NO_2_), ozone and various toxic compounds.^13–17^ Vulnerable populations, including children,^18–22^ older people, pregnant women^23–25^ and individuals with pre-existing health conditions,^15,26^ are particularly susceptible to the adverse health effects associated with smoke exposure.^27–29^

Recent studies indicate a direct correlation between the frequency and intensity of wildfires and rising global temperatures, exacerbated by extreme weather events such as prolonged heatwaves and droughts.^30–35^ The Intergovernmental Panel on Climate Change (IPCC) projects continued increases in global temperatures, which are likely to enhance conditions conducive to wildfires, thereby intensifying their public health impacts.^3^ This escalating threat establishes a cycle of environmental and health crises that demands urgent attention.^36^

The health risks associated with wildfire smoke exposure are well documented, encompassing acute respiratory issues, cardiovascular complications and exacerbation of chronic conditions.^37–39^ Moreover, the long-term consequences of recurrent smoke exposure remain underexplored, highlighting a significant gap in the existing literature.^16^ Understanding the socioeconomic factors that exacerbate these health risks is also critical, as they play a pivotal role in shaping community resilience.^40–44^

The intricate relationship between climate change, wildfires and public health needs coordinated responses from a diverse array of stakeholders, including public health experts, researchers and policymakers.^45,46^ Given the severity of wildfires and their implications for public health, there is an urgent need for comprehensive interventions that prioritise preparedness, response and the safeguarding of vulnerable populations.^47–49^

This bibliometric analysis explores the growing body of research on the health impacts of wildfires. It examines trends in publications, geographical distribution, collaboration networks, key researchers and institutions, as well as funding sources. To explore the current state of research on wildfire and health, this study seeks to investigate how wildfire and health research is being produced across the globe. Considering the need for a structured assessment to be carried out of scientific production addressing the research topic, the objective of this paper is to analyse the scientific publications encompassing wildfire and health research. As such, the following research questions were proposed to guide the study: (1) Which are the main countries and research institutes publishing on this topic? (2) Which authors have published the most? (3) Which are the leading scientific journals addressing this topic? The analysis will focus on publication and citation trends, geographical location, authors and institutions, journals and publishers, funding agencies, research areas and keyword analysis.

## Methods

To address the proposed questions, this study used a bibliometric analysis of the literature on wildfires and health. This methodology was chosen to evaluate specific outputs, drawing on the framework established by Ellegaard and Wallin.^50^ Bibliometric research, often referred to as the ‘science of science’,^51^ offers a quantitative approach to analysing large datasets, enabling researchers to assess the current state of research, identify emerging trends and uncover opportunities for future collaboration.^52^

Data for this study were extracted from the Web of Science (WoS) Core Collection, a highly reputable database.^53,54^ The WoS Core Collection (Clarivate; https://webofscience.help.clarivate.com/Content/wos-core-collection/wos-core-collection.htm) is highly suitable for bibliometric studies due to its comprehensive coverage of >22 000 peer-reviewed journals across 254 subject areas, spanning the sciences, social sciences, arts and humanities. It includes >2.3 million cited references and >95 million records, providing researchers with extensive tools for citation analysis. WoS allows for tracking citation patterns and assessing the academic impact of publications, making it an essential resource for evaluating research influence globally. Its robust indexing and citation metrics make it one of the most authoritative and reliable databases for bibliometric analysis.^55^ Unlike scoping or systematic reviews, which require broader content coverage, including grey literature and non-peer-reviewed sources, bibliometric analysis focus on citation trends and scholarly impact, making WoS the optimal choice. Therefore, the use of WoS in this study is justified, as it ensures a rigorous and reliable assessment of research influence across multiple disciplines.

The search was conducted until 23 September 2024, focusing exclusively on original research articles published in English. The following search string was used: TS=((‘human health’) AND (‘forest fire*’ OR ‘bush fire*’ OR ‘bushfire*’ OR ‘wildfire*’ OR ‘wild fire’ OR ‘wildland fire*’ OR ‘wild land fire*’)) and Article (Document Types) and English (Languages). Bibliographic information, including titles, authors, institutions, countries, abstracts and keywords, were exported from WoS for further analysis and visualisation using VOSviewer. VOSview (Leiden University; Leiden; Netherlands, https://www.vosviewer.com/)er is widely recognised in bibliometric research for its ability to construct and visualise relationships within bibliometric data, displaying links and clusters through different colours.^51,56,57^

Quantitative data from the reference articles were used to create graphs, tables, bibliographic networks and textual data networks. A comprehensive data network encompassing all the articles was constructed using VOSviewer software. To capture the full scope of wildfire and health literature, the term-based search query developed was applied in the advanced search section of WoS, targeting key terms exclusively in the titles, keywords and abstracts of indexed articles. The complete bibliographic details of all items were exported as text files.

## Results

The comprehensive search yielded 483 original research articles on wildfire and health research, published from 1 January 2000 to 23 September 2024. This figure (N=483) offers an approximate estimation of the extent of the existing literature at that time (Table 1).

**Table 1.** Summary of characteristics of included research articles

	N (%) of research articles
**Exposure**	
Wildfire	483 (100.0%)
**Year of publication**	
2000	1 (0.2%)
2001	2 (0.4%)
2002	1 (0.2%)
2003	3 (0.6%)
2004	3 (0.6%)
2005	1 (0.2%)
2006	1 (0.2%)
2007	4 (0.8%)
2008	5 (1.0%)
2009	4 (0.8%)
2010	8 (0.8%)
2011	5 (1.0%)
2012	4 (0.8%)
2013	7 (1.4%)
2014	13 (2.7%)
2015	14 (2.9%)
2016	14 (2.9%)
2017	24 (5.0%)
2018	30 (6.2%)
2019	38 (7.9%)
2020	48 (9.9%)
2021	64 (13.3%)
2022	66 (13.7%)
2023	71 (14.7%)
2024	51 (10.6%)
Geographic regions*^	
Africa	17 (2.2%)
Asia	149 (19.0%)
Europe	235 (30.0%)
Latin America and the Caribbean	41 (5.2%)
North America	285 (36.4%)
Oceania	57 (7.3%)
Top publishing countries*	
USA	248 (51.3%)
Australia	52 (10.8%)
People’s Republic of China	51 (10.6%)
Canada	34 (7.0%)
England	28 (5.8%)
Italy	25 (5.2%)
Germany	23 (4.8%)
Brazil	19 (3.9%)
India	18 (3.7%)
Japan	15 (3.1%)
The Netherlands	15 (3.1%)
**Top publishing authors**	
Tong D	8 (1.7%)
Price OF	7 (1.4%)
Johnston FH	6 (1.2%)
Randerson JT	6 (1.2%)
Williamson GJ	5 (1.0%)
Russell AG	5 (1.0%)
**Top publishing affiliations**	
Colorado State University	21 (4.3%)
University of Washington	17 (3.5%)
National Aeronautics Space Administration (NASA)	17 (3.5%)
United States Environmental Protection Agency (US EPA)	17 (3.5%)
National Oceanic and Atmospheric Administration (NOAA)	16 (3.3%)
University of Tasmania	15 (3.1%)
University of Colorado	15 (3.1%)
United States Forest Service	15 (3.1%)
**Top journals**	
*Science of the Total Environment*	28 (5.8%)
*Atmospheric Environment*	19 (3.9%)
*Atmosphere*	18 (3.7%)
*Atmospheric Chemistry and Physics*	18 (3.7%)
*Environmental Research Letters*	15 (3.1%)
*Journal of Geophysical Research: Atmospheres*	12 (2.5%)
*Environmental Pollution*	12 (2.5%)
**Top publishers**	
Elsevier	144 (29.8%)
MDPI	59 (12.2%)
Springer Nature	51 (1.6%)
American Geophysical Union	33 (6.8%)
Copernicus Gesellschaft Mbh	29 (6.0%)
Wiley	22 (4.6%)
Taylor & Francis	19 (3.9%)
IOP Publishing Ltd	15 (3.1%)
American Chemical Society	10 (2.1%)
Csiro Publishing	10 (2.1%)
**Top funding agencies**	
National Science Foundation (NSF)	40 (8.3%)
National Natural Science Foundation of China (NSFC)	28 (5.8%)
NASA	27 (5.6%)
United States Department of Health Human Services	25 (5.2%)
National Institutes of Health (NIH USA)	24 (5.0%)
NOAA USA	19 (3.9%)
European Union (EU)	18 (3.7%)
Unites States Department of Energy (DOE)	18 (3.7%)
National Institute of Environmental Health Sciences (NIEHS)	14 (2.9%)
US EPA	14 (2.9%)
**Top research areas**	
Environmental Sciences Ecology	282 (58.4%)
Meteorology Atmospheric Sciences	139 (28.8%)
Geology	48 (9.9%)
Public Environmental Occupational Health	48 (9.9%)
Engineering	36 (7.5%)
Forestry	29 (6.0%)
Science Technology Other Topics	28 (5.8%)
Water Resources	18 (3.7%)
Remote Sensing	16 (3.3%)
Toxicology	15 (3.1%)

*Research articles may be counted more than once if there were multiple options.

^According to https://population.un.org/wpp/definition-of-regions.

### Publication and citation trends

**Figure 1.**
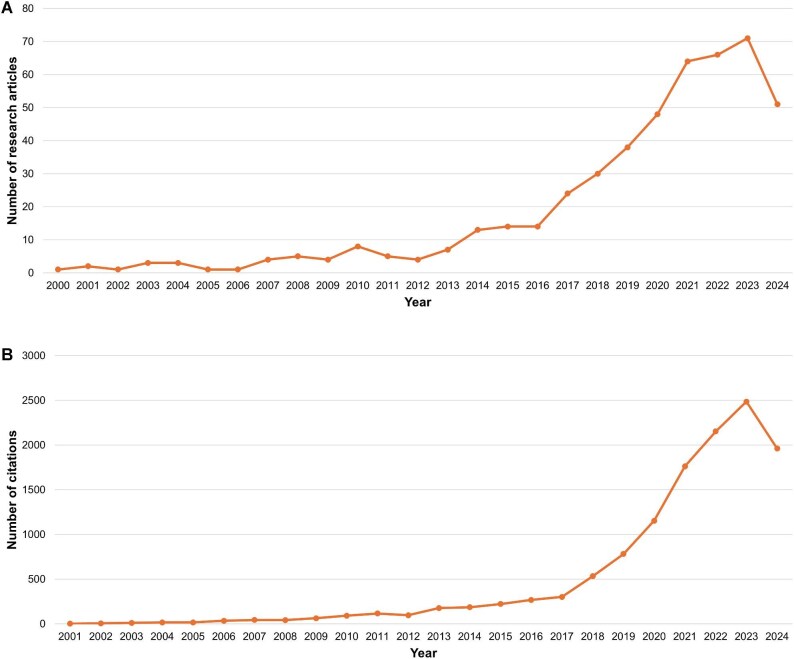
(A) and (B) Distribution of annual publications and citations.

The earliest article retrieved from the WoS Core Collection database was published in 2000 (Table 1). Since then, the volume of publications in the field has steadily increased, as reflected in both the number of articles and their citations (Figure 1A,B). The number of publications and citations has shown a particularly marked rise since 2016. With a compound annual growth rate of 16.0%, 2023, saw so far, the highest yet number of publications (n=71) and citations (n=2486) (Figure 1B).

### Geographical distribution

**Figure 2.**
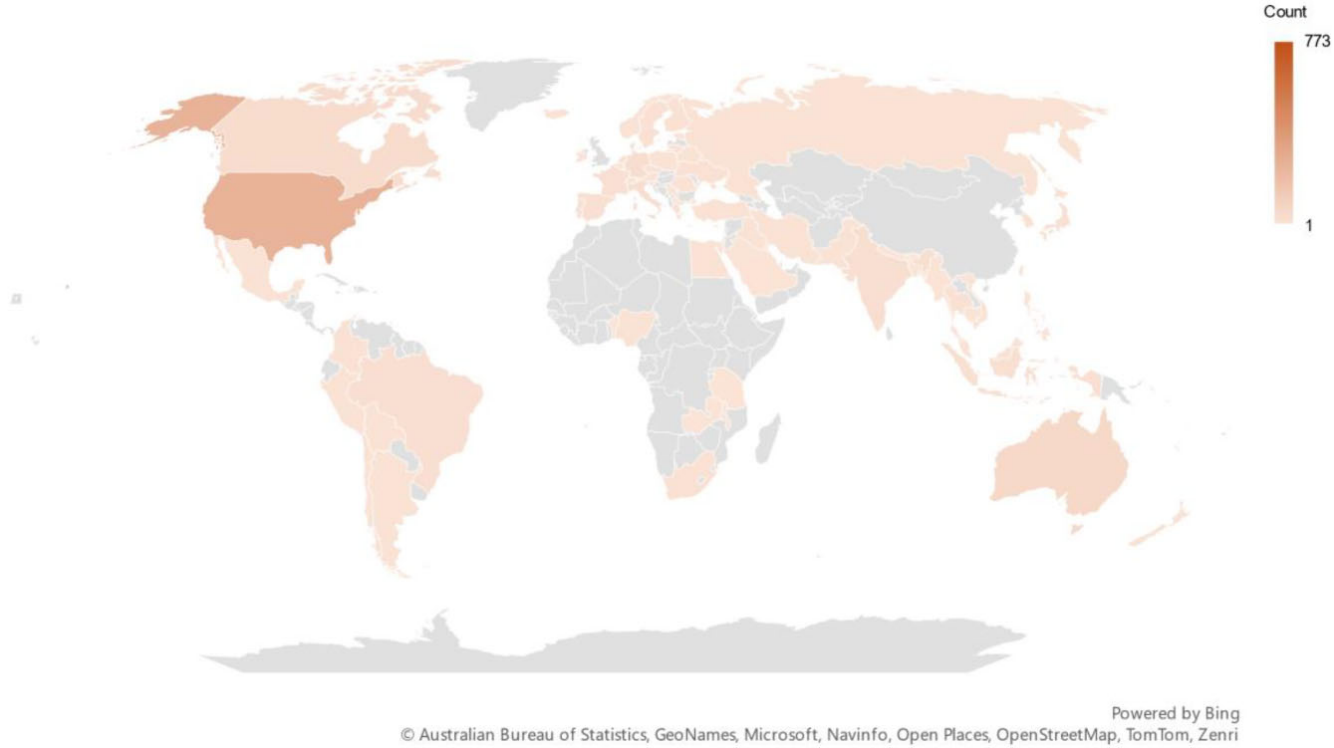
Distribution of geographical locations.

Research on wildfire and health has seen significant regional disparities. Most publications originated from North America (36.4%), followed by Europe (30.0%) and Asia (19.0%) (Table 1). By contrast, Oceania, Latin America and the Caribbean, and Africa, accounted for only 7.3%, 5.2% and 2.2% of the total publications, respectively. The number of research articles from North America is substantially higher than those of other regions at 285 articles (Table 1). Seventy-six countries have published at least one article devoted to wildfire and health research. The USA leads with 248 articles, considerably higher than other countries, followed by Australia (n=52), China (n=51) and Canada (n=34) (Figure 2). In addition, the top 10 countries in publications also included England (n=28), Italy (n=25), Germany (n=23), Brazil (n=19), India (n=18), the Netherlands (n=15) and Japan (n=15). Total citations in the USA (6607), Australia (2045) and China (1089) further emphasise their impact in this research area. The top 10 countries in citations also included Canada (n=871), Finland (n=595), Austria (n=569), Italy (n=511), Germany (n=508) and Spain (n=477) (Table 1). Interestingly, despite producing fewer articles (n=28), England's higher citation count (1049) ranks it fourth in the field, surpassing Canada.

**Figure 3.**
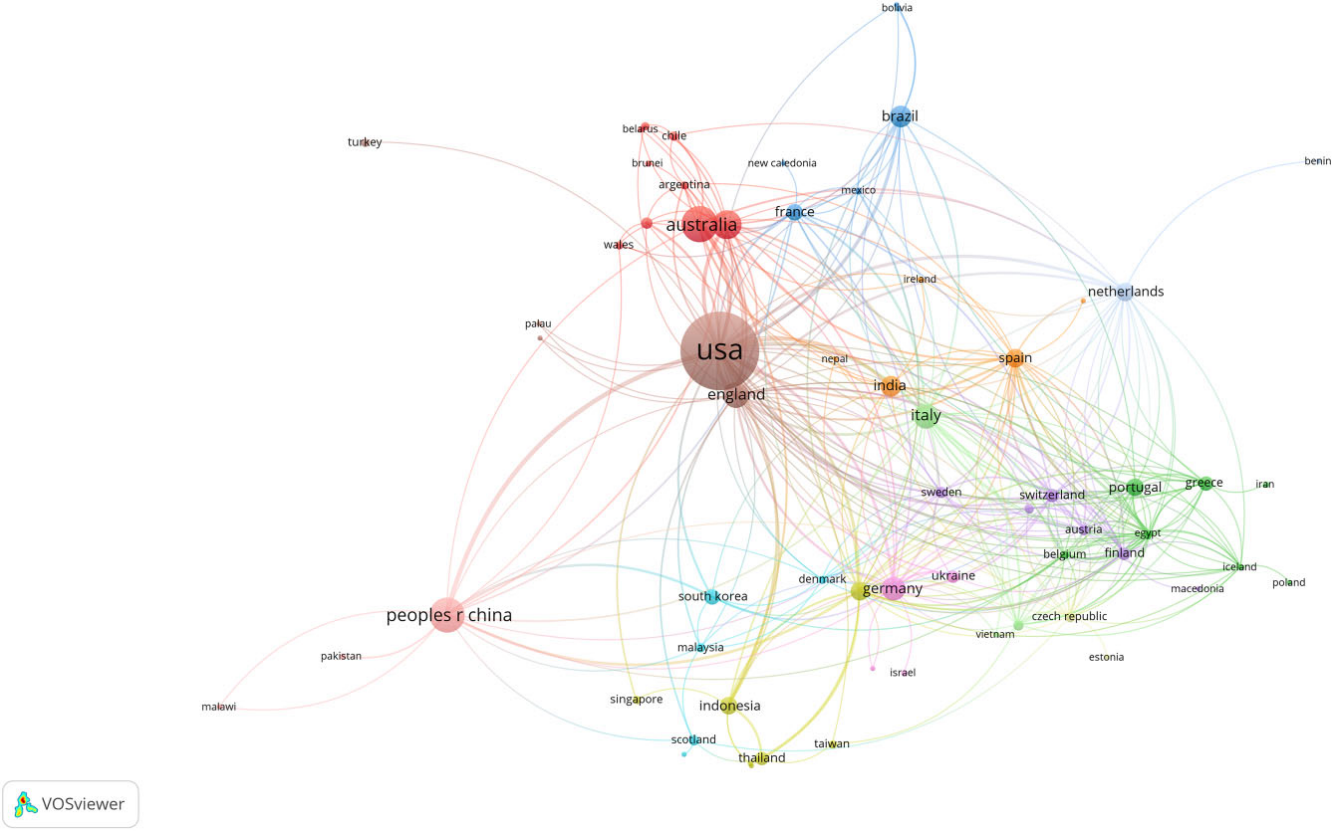
Visualisation of the most connected countries.

Figure 3 visualises international collaboration network within the field, where a node represents a country, while the connections between nodes indicate coauthorship among countries. The thickness of each connection correlates with the number of wildfire and health articles in which two countries are listed together, also known as link strength. By aggregating the strengths of the links associated with each node, a metric called total link strength (TLS) is derived. This metric reflects the overall extent of a country's coauthorship activities with other countries. In the map shown in Figure 3, the size of each node corresponds to this metric, meaning that larger nodes signify countries that have collaborated more frequently with other countries within the network. Additionally, the diversity of a country's coauthorship can be represented by the number of links extending from their respective node. Countries that have frequently collaborated on wildfire and health publications can create a cluster. The USA had the highest TLS (TLS=144), followed by Italy (TLS=68), England (TLS=65) and Germany (TLS=49) (Supplementary Table 1). Out of 76 countries, 74 meet the criteria of at least one publication and one citation. For each of the 74 countries, the total strength of the coauthorship links with other countries was calculated. The countries with the greatest TLS were selected. Some of the 74 countries in the network are not connected to each other. The largest set of connected countries consists of 69, highlighting the USA as the primary hub for collaborations in this research area. The analysis includes only these countries.

### Authors and institutions

A total of 2605 authors contributed to the field with at least one article from 2000 to 2024, with the top six authors publishing 37 articles (Table 1). The most prolific author was Tong D (n=8), followed by Prince OF (n=7), Johnston FH (n=6) and Randerson JT (n=6). In terms of citation impact, Johnston FH, Bell ML, Kinney PL and Abrahamson MJ each received >400 citations, significantly surpassing those of other researchers.

**Figure 4.**
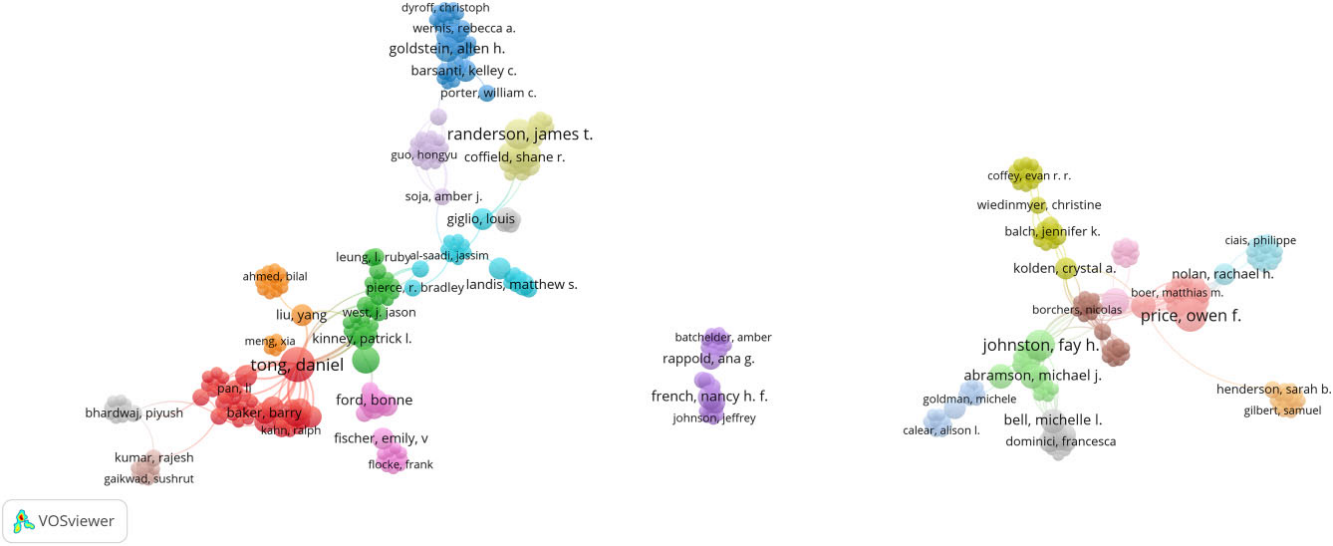
Visualisation of the most connected authors.

The collaboration patterns among authors were examined using VOSviewer. Of the 2605 authors, 2369 meet the threshold of at least one publication and citation. For each of the 2369 authors, the total strength of coauthorship links with other authors was calculated. The authors with the greatest TLS were selected. Some of the authors in the network are not connected to each other. The largest set of connected authors consists of 401 authors. The analysis will include only these authors. Figure 4 depicts the coauthorship network. Each node in the network represents an author, while the connections between nodes indicate coauthorship among authors. The thickness of each connection correlates with the number of wildfire and health articles in which two authors are listed together as coauthors, also known as link strength. By aggregating the strengths of the links associated with each node, a metric called total link strength is derived. This metric reflects the overall extent of an author's coauthorship activities with other authors in the network. In the map shown in Figure 4, the size of each node corresponds to this metric, meaning that larger nodes signify authors who have collaborated more frequently with other authors within the network. Additionally, the diversity of an author's coauthorship can be represented by the number of links extending from their respective node. Authors who have frequently collaborated on wildfire and health publications can create a cluster. Daniel Tong had the highest TLS (TLS=95), followed by Williamson GJ (TLS=59) and Johnston FH (TLS=40) (Supplementary Table 2).

**Figure 5.**
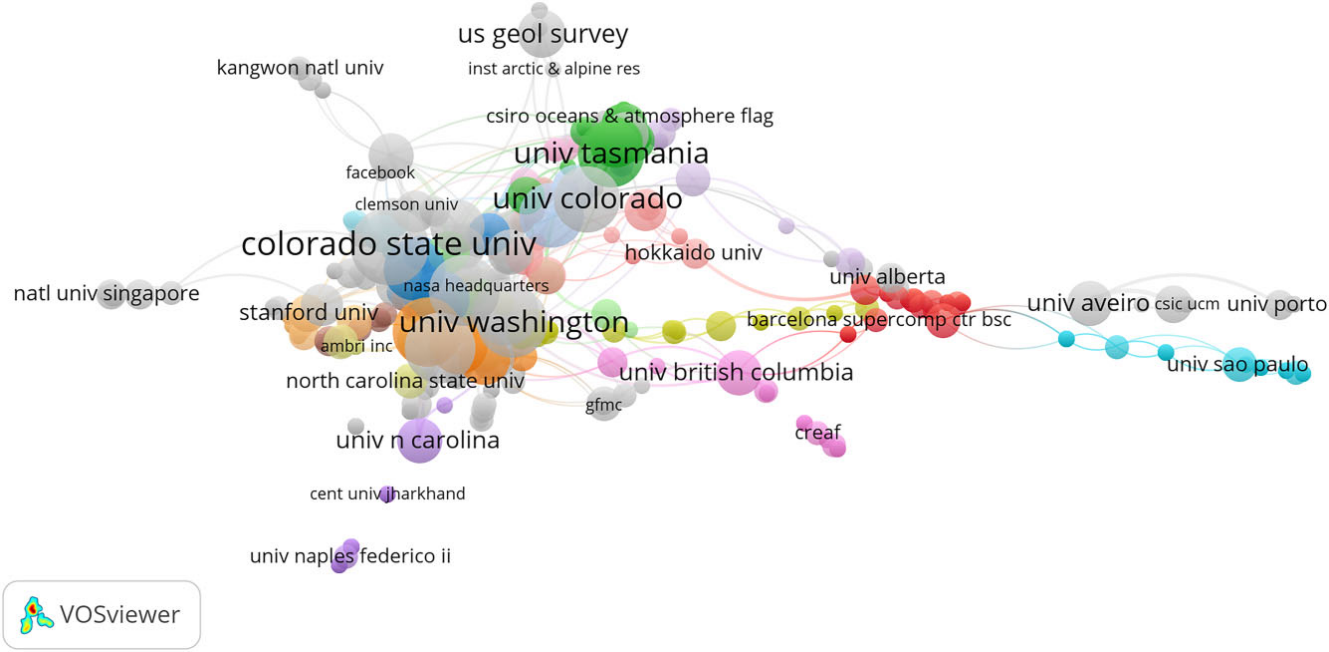
Visualisation of the most connected institutions.

Institutional analysis identified 908 institutions involved in the field and having published at least one article. Colorado State University led with 21 articles, followed by the University of Washington (n=17), National Aeronautics Space Administration (NASA) (n=17), United States Environmental Protection Agency (US EPA) (n=17) and National Oceanic and Atmospheric Administration (NOAA) (n=16). This pattern reveals that a significant proportion of the knowledge is produced with contributions from the USA. Interestingly, the University of Tasmania had the highest citation count (C=955), followed by Colorado State University (C=775). The Finnish Environmental Institute (SYKE) and the International Institute for Applied Systems Analysis (IIASA) had only one article each but recorded the eighth highest average number of citations (C=478). Of the 908 institutions, 855 meet the threshold of at least one publication and citation. For each of the 855 institutions, the total strength of coauthorship links with other institutions were calculated. The institutions with the greatest TLS were selected. Some of the 855 institutions in the network are not connected to each other. The largest set of connected countries consists of 613 countries. The analysis will include only these countries. Collaboration analysis showed that four out of five of the most influential institutions are based in the USA. This indicates that only a limited number of USA institutions are actively leading in this area of research, with the University of Washington having the highest TLS (TLS=82), followed by NASA (TLS=80), NOAA (TLS=69) and the University of Colorado (TLS=65) (Supplementary Table 3). Figure 5 illustrates the collaboration network among institutions in wildfire and health literature. Each node in the network represents an institution, while the connections between nodes indicate coauthorship among institutions. The thickness of each connection correlates with the number of wildfire and health articles in which two institutions are listed together, also known as link strength. By aggregating the strengths of the links associated with each node, a metric called total link strength is derived. This metric reflects the overall extent of an institution's coauthorship activities with other institutions in the network. In the map shown in Figure 5, the size of each node corresponds to this metric, meaning that larger nodes signify institutions that have collaborated more frequently with other institutions within the network. Additionally, the diversity of an institution's coauthorship can be represented by the number of links extending from their respective node. Institutions that have frequently collaborated on wildfire and health publications can create a cluster.

### Journals and publishers

The bibliometric analysis of journals not only indicates their scholarly impact within the field but also helps researchers in making submission decisions. A total of 219 journals have published at least one article on wildfire and health research. This research has been published in journals spanning various academic fields. The most prolific journal was *Science of the Total Environment* with 28 articles, followed by *Atmospheric Environment* (n=19), *Atmosphere* (n=18) and *Atmospheric Chemistry and Physics* (n=18). *Atmospheric Chemistry and Physics* led in citations (C=886), with citations per article of 49.2% (Supplementary Table 4). Of the 219 journals, 202 meet the threshold of at least one publication and citation. For each of the 202 journals, the total strength of the citation links with other journals was calculated. The journals with the greatest TLS were selected. Some of the 202 journals in the network are not connected to each other. The largest set of connected journals consists of 113 journals. The analysis will include only these journals. Of the top journals, *Science of the Total Environment* had the highest TLS (TLS=99) (Science Direct), followed by *Environmental Research Letters* (TLS=39) (IOP), Atmosphere (TLS=37) (MDPI) and *Atmospheric Chemistry and Physics* (TLS=37) (European Geosciences Union) (Supplementary Table 4). Elsevier (n=144, 29.8%), MDPI (n=59, 12.2%) and Springer Nature (n=51, 10.6%) were the leading publishers (Table 1).

### Funding agencies

The five most mentioned funding agencies were primarily based in the USA, with the National Science Foundation leading (n=40, 8.3%), followed by the National Natural Science Foundation of China (n=28, 5.8%), NASA (n=27, 5.6%), the United States Department of Health Human Services (n=25, 5.2%) and the National Institutes of Health USA (n=24, 5.0%) (Table 1). Collectively, USA agencies funded 135 of the total research articles (Table 1).

### Research areas

**Figure 6.**
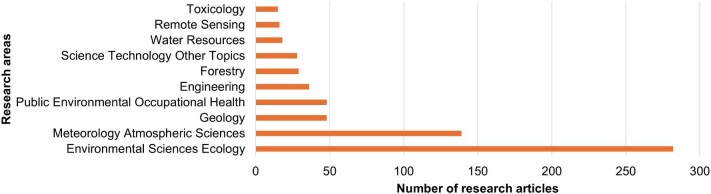
Top 10 research areas in terms of wildfire and health research.

In the WoS Core Database, each article is categorised into specific research areas. Figure 6 illustrates the main categories of publications, along with the corresponding number and percentage of articles in each category. The largest share of publications fell under ‘Environmental Sciences Ecology’ (n=256; 53.0%), followed by ‘Meteorology Atmospheric Sciences’ (n=139; 28.8%) (Table 1).

### Keyword analysis

The most frequently occurring keywords included ‘wildfire(s)’ (n=90), ‘climate change’ (n=52), ‘air quality’ (n=46), ‘air pollution’ (n=37), ‘biomass burning’ (n=33), ‘particulate matter’ (n=30), ‘human health’ (n=29), ‘smoke’ (n=21), ‘forest fire’ (n=16) and ‘remote sensing’ (n=15).

**Figure 7.**
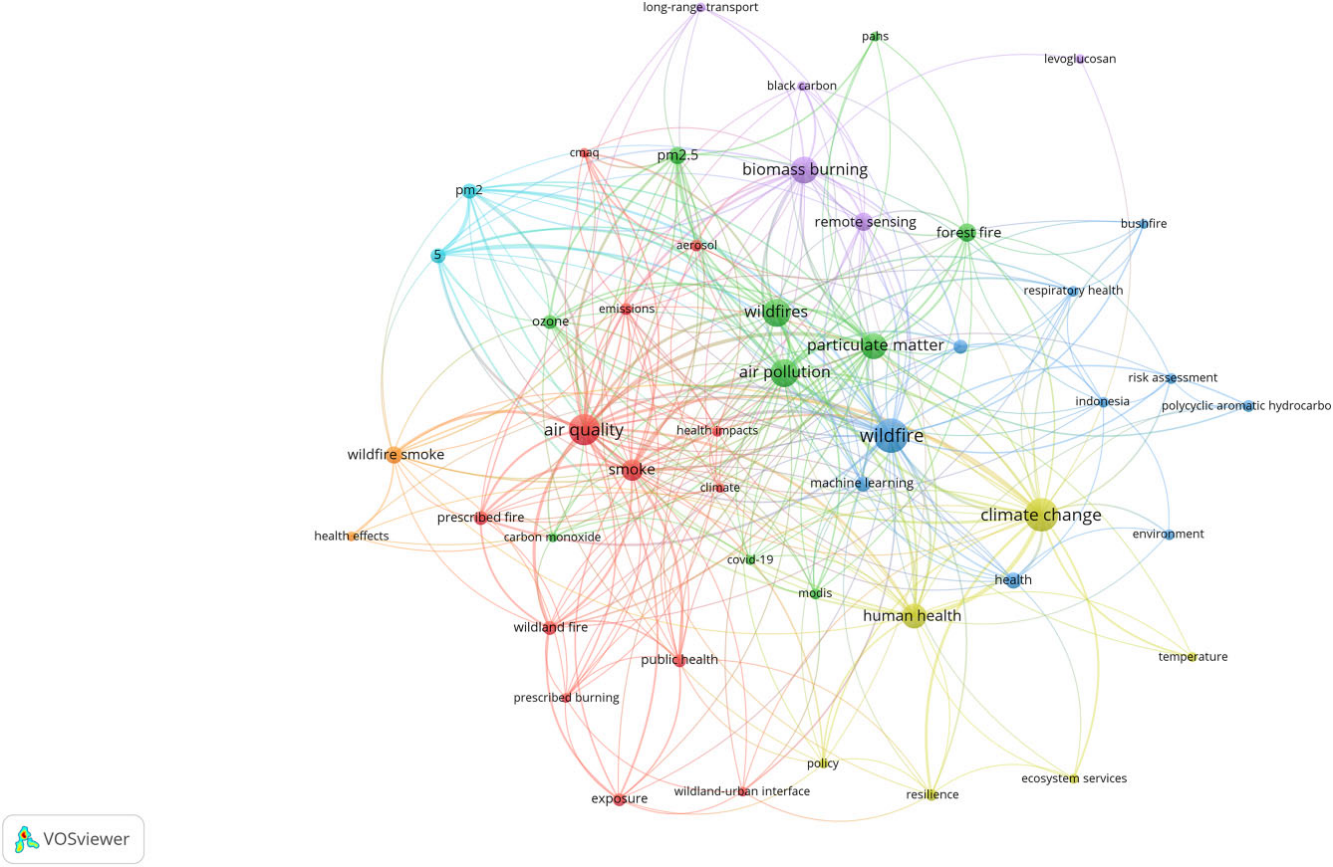
Co-occurrence network among keywords in wildfire and health research.

VOSviewer was used to map the frequency of keywords, with the size of the nodes and fonts corresponding to their occurrence rate, as illustrated in Figure 7. Of the 1347 keywords, 48 meet the threshold of at least five occurrences. For each of the 48 keywords, the total strength of co-occurrence links with other keywords was calculated. ‘Air quality’ showed the highest TLS (TLS=113), followed by ‘wildfire’ (TLS=81), ‘climate change’ (TLS=79) and ‘air pollution’ (TLS=73) (Supplementary Table 5). The keywords with the greatest TLS were selected. Figure 7 illustrates the co-occurrence network among keywords. Each node in the network represents a keyword, while the connections between nodes indicate co-occurrence among keywords. The thickness of each connection correlates with the number of wildfire and health articles in which two keywords are listed together, also known as link strength. By aggregating the strengths of the links associated with each node, a metric called total link strength is derived. This metric reflects the overall extent of a keyword's co-occurrence with other keywords in the network. In the map shown in Figure 7, the size of each node corresponds to this metric, meaning that larger nodes signify keywords that have co-occurred more frequently with other keywords within the network. Additionally, the diversity of a keyword's co-occurrence can be represented by the number of links extending from their respective node. Keywords that have frequently co-occurred on wildfire and health publications can create a cluster.

The analysis identified 48 keywords organised in seven major clusters, each reflecting distinct themes. Clusters are further explained in Table 2. Cluster 1 mainly focuses on the effects of wildfires on public health, while cluster 2 focuses on air pollution caused by wildfires, and cluster 3 highlights environmental factors and health outcomes related to wildfires. The co-occurrence network, shown in Figure 7, highlights the interconnectedness of these themes, with clusters representing different focus areas within the literature.

**Table 2.** Clusters of keywords based on authors’ keywords

Keyword cluster	Number of keywords in cluster	Keywords
Cluster 1 (red)	13	‘aerosol’; ‘air quality’; ‘climate’; ‘cmaq’; ‘emissions’; ‘exposure’; ‘health impacts’; ‘prescribed burning’; ‘prescribed fire’; ‘public health’; ‘smoke’; ‘wildland fire’; ‘wildland-urban interface’
Cluster 2 (green)	10	‘air pollution’; ‘carbon monoxide’; ‘covid-19’; ‘forest fire’; ‘modis’; ‘ozone’; ‘pahs’; ‘particulate matter’; ‘pm2.5’; ‘wildfires’
Cluster 3 (blue)	10	‘bushfire’; ‘environment’; ‘forest fires’; ‘health’; ‘indonesia’; ‘machine learning’; ‘polycyclic aromatic hydrocarbons’; ‘respiratory health’; ‘risk assessment’; ‘wildfire’
Cluster 4 (yellow)	6	‘climate change’; ‘ecosystem services’; ‘human health’; ‘policy’; ‘resilience’; ‘temperature’
Cluster 5 (purple)	5	‘biomass burning’; ‘black carbon’; ‘levoglucosan’; ‘long-range transport’; ‘remote sensing’
Cluster 6 (aquamarine)	2	‘5’; ‘pm2’
Cluster 7 (orange)	2	‘health effects’; ‘wildfire smoke’

## Discussion

This bibliometric analysis demonstrates the growing relevance of research linking wildfire and health, as shown by the increase in both publications and citations over the past two decades. Since 2000, there has been a steady rise in research, with a marked acceleration after 2016, reflecting the increasing global recognition of wildfires as a public health threat, driven by factors such as climate change and population growth. The compound annual growth rate of publications (16.0%) and a notable peak in 2023 (71 publications and 2486 citations) suggest sustained interest, likely tied to the intensifying frequency and severity of wildfires worldwide.

Geographically, research output is concentrated in North America,^17,58,59^ Europe^33,38^ and Asia, with under-representation in regions such as Oceania,^28,46^ Latin America and Africa, despite their vulnerability to wildfires. Expanding research efforts in these regions is crucial for a more comprehensive understanding of wildfire health impacts. The USA leads both in the number of publications and citations, reflecting its strong research infrastructure. This disparity points to a need for greater focus on wildfire impacts in less studied regions to ensure more global insights.

Collaboration networks show the interdisciplinarity and international nature of wildfire health research, with the USA playing a central role in facilitating cross-border collaborations. European countries such as Italy, England and Germany also show strong research ties. However, gaps in collaboration, particularly with lower-income countries, suggest missed opportunities for wider research engagement. The analysis also identifies key contributors, with authors such as Daniel Tong and institutions such as Colorado State University and NASA leading the field. However, the dominance of US-based institutions highlights a concentration of intellectual leadership, underscoring the need for more diverse institutional participation.

Journal analysis reveals that a small number of journals, such as *Science of the Total Environment* and *Atmospheric Chemistry and Physics*, serve as major platforms for wildfire health research. The interdisciplinary nature of this field is evident, although the concentration in specific journals may limit visibility in broader health policy discussions.

Keyword analysis identifies dominant themes, such as the effects of wildfire air pollution and broader environmental health impacts. However, there appears to be a relative under-representation of health-specific terms, suggesting that health outcomes are not explored as extensively as environmental aspects. More research into the long-term health effects of wildfire exposure is essential for a comprehensive understanding of public health risks.

The role of funding agencies in shaping research priorities largely determines the breadth and focus of research efforts. Funding analysis shows that US agencies such as the National Science Foundation and National Institutes of Health are key supporters of wildfire health research. However, the concentration of funding in certain regions highlights potential inequities. Expanding funding opportunities to under-represented regions is critical to address knowledge gaps and ensure global preparedness for wildfire-related health risks and impacts.

Wildfire and health research is rapidly growing, with significant contributions from North America and Europe. However, research gaps remain, particularly in regions vulnerable to wildfires but under-represented in the literature. One of the study limitations is the exclusion of non-English publications, which may have affected the geographical representation of wildfire and health research. However, our decision was based on ensuring consistency in data extraction and interpretation, particularly given that bibliometric tools such as VOSviewer primarily support English-language processing.

In response to the above, we propose several recommendations, which include advocating for interdisciplinary funding calls, enhancing international research collaboration and increasing engagement with policy stakeholders to direct resources toward wildfire-related health research.

Future efforts should promote international collaboration, broader institutional involvement and a stronger focus on health outcomes. Equitable distribution of resources is essential to ensure global needs are addressed, especially as climate change drives more frequent wildfire events.

## Conclusion

This study highlights the increasing significance of wildfire and health research and identifies key gaps that must be addressed. Expanding research participation, promoting international collaboration and increasing the focus on health-specific outcomes will better equip the field to address the escalating public health challenges posed by wildfires.

## Supplementary Material

ihaf032_Supplemental_Files

## Data Availability

The datasets generated during and/or analysed during the current study are publicly available in the Web of Science Core Collection repository.
