# Supplementary material for: The state of wildfire and health research: emerging trends, challenges and gaps
Source: Int Health. 2025 Apr 8;17(6):922–33. doi: 10.1093/inthealth/ihaf032 (PMC12585580; doi:10.1093/inthealth/ihaf032)
Supplement: ihaf032_Supplemental_Files [file ihaf032_supplemental_files.zip › Supplementary Table 1.docx]

**Supplementary Table 1.** Top 10 countries with the greatest total link strength

| Rank | Country | P | C | TLS |
| --- | --- | --- | --- | --- |
| 1 | USA | 248 | 6607 | 144 |
| 2 | Italy | 25 | 511 | 68 |
| 3 | England | 28 | 1049 | 65 |
| 4 | Germany | 23 | 508 | 49 |
| 5 | Japan | 15 | 273 | 48 |
| 6 | Spain | 14 | 477 | 47 |
| 7 | Netherlands | 15 | 321 | 42 |
| 8 | China | 51 | 1089 | 42 |
| 9 | Switzerland | 8 | 181 | 39 |
| 10 | Canada | 34 | 871 | 37 |

*P: number of publications; C: number of citations; TLS: total link strength
