# Supplementary material for: The state of wildfire and health research: emerging trends, challenges and gaps
Source: Int Health. 2025 Apr 8;17(6):922–33. doi: 10.1093/inthealth/ihaf032 (PMC12585580; doi:10.1093/inthealth/ihaf032)
Supplement: ihaf032_Supplemental_Files [file ihaf032_supplemental_files.zip › Supplementary Table 2.docx]

**Supplementary Table 2.** Top 11 most influential authors

| Rank | Author | Country | C | P | AC | TLS |
| --- | --- | --- | --- | --- | --- | --- |
| 1 | Tong, D | USA | 179 | 8 | 22.4 | 95 |
| 2 | Williamson, GJ | Australia | 305 | 5 | 61 | 59 |
| 3 | Johnston FH | Australia | 691 | 6 | 115.2 | 40 |
| 4 | Lee, P | USA | 85 | 3 | 28.3 | 38 |
| 5 | Liu, Y | USA | 276 | 3 | 92 | 38 |
| 6 | Nolan, RH | Australia | 72 | 3 | 24 | 38 |
| 7 | Saylor, R | USA | 49 | 3 | 16.3 | 38 |
| 8 | Bowman, DMJS | USA | 233 | 4 | 58.3 | 37 |
| 9 | Baker, B | USA | 20 | 2 | 10 | 36 |
| 10 | Randerson, JT | USA | 137 | 6 | 22.8 | 36 |
| 11 | Tang, Y | USA | 20 | 3 | 6.7 | 36 |

*P: number of publications; C: number of citations; AC: average citations; TLS: total link strength
