# Supplementary material for: The state of wildfire and health research: emerging trends, challenges and gaps
Source: Int Health. 2025 Apr 8;17(6):922–33. doi: 10.1093/inthealth/ihaf032 (PMC12585580; doi:10.1093/inthealth/ihaf032)
Supplement: ihaf032_Supplemental_Files [file ihaf032_supplemental_files.zip › Supplementary Table 3.docx]

**Supplementary Table 3.** Top 11 most influential institutions

| Rank | Institution | Country | C | P | AC | TLS |
| --- | --- | --- | --- | --- | --- | --- |
| 1 | University of Washington | USA | 542 | 17 | 31.9 | 82 |
| 2 | NASA | USA | 387 | 17 | 22.8 | 80 |
| 3 | NOAA | USA | 284 | 16 | 17.8 | 69 |
| 4 | University of Tasmania | Australia | 955 | 15 | 63.7 | 67 |
| 5 | University of Colorado | USA | 657 | 15 | 43.8 | 65 |
| 6 | Chinese Academy of Sciences | China | 261 | 14 | 18.6 | 59 |
| 7 | George Mason University | USA | 155 | 11 | 14.1 | 59 |
| 8 | Colorado State University | USA | 775 | 21 | 36.9 | 55 |
| 9 | University of North Carolina | USA | 167 | 7 | 23.9 | 55 |
| 10 | University of California Berkeley | USA | 142 | 13 | 10.9 | 52 |
| 11 | University of New South Wales | Australia | 111 | 6 | 18.5 | 52 |

*P: number of publications; C: number of citations; AC: average citations; TLS: total link strength
