# Supplementary material for: The state of wildfire and health research: emerging trends, challenges and gaps
Source: Int Health. 2025 Apr 8;17(6):922–33. doi: 10.1093/inthealth/ihaf032 (PMC12585580; doi:10.1093/inthealth/ihaf032)
Supplement: ihaf032_Supplemental_Files [file ihaf032_supplemental_files.zip › Supplementary Table 4.docx]

**Supplementary Table 4.** Top 10 most active journals

| Rank | Journal | P | C | TLS |
| --- | --- | --- | --- | --- |
| 1 | Science of the Total Environment | 28 | 841 | 99 |
| 2 | Environmental Research Letters | 15 | 381 | 39 |
| 3 | Atmosphere | 18 | 169 | 37 |
| 4 | Atmospheric Chemistry and Physics | 18 | 886 | 37 |
| 5 | Geohealth | 7 | 277 | 35 |
| 6 | Journal of Geophysical Research: Atmospheres | 12 | 235 | 35 |
| 7 | Atmospheric Environment | 19 | 437 | 30 |
| 8 | Climatic Change | 3 | 234 | 25 |
| 9 | Nature Communications | 1 | 247 | 25 |
| 10 | International Journal of Wildland Fire | 8 | 92 | 20 |

*P: number of publications; C: number of citations; TLS: total link strength
