# Supplementary material for: The state of wildfire and health research: emerging trends, challenges and gaps
Source: Int Health. 2025 Apr 8;17(6):922–33. doi: 10.1093/inthealth/ihaf032 (PMC12585580; doi:10.1093/inthealth/ihaf032)
Supplement: ihaf032_Supplemental_Files [file ihaf032_supplemental_files.zip › Supplementary Table 5.docx]

**Supplementary Table 5.** Top 11 most co-occurring keywords

| Rank | Keywords | Occurrences | TLS |
| --- | --- | --- | --- |
| 1 | ‘air quality’ | 46 | 113 |
| 2 | ‘wildfire’ | 55 | 81 |
| 3 | ‘climate change’ | 52 | 79 |
| 4 | ‘air pollution’ | 37 | 73 |
| 5 | ‘particulate matter’ | 30 | 67 |
| 6 | ‘smoke’ | 21 | 62 |
| 7 | ‘biomass burning’ | 33 | 52 |
| 8 | ‘human health’ | 29 | 52 |
| 9 | ‘wildfires’ | 35 | 52 |
| 10 | ‘5’ | 11 | 39 |
| 11 | ‘pm2’ | 11 | 39 |

*TLS: total link strength
